# A VLBI experiment using a remote atomic clock via a coherent fibre link

**DOI:** 10.1038/srep40992

**Published:** 2017-02-01

**Authors:** Cecilia Clivati, Roberto Ambrosini, Thomas Artz, Alessandra Bertarini, Claudio Bortolotti, Matteo Frittelli, Filippo Levi, Alberto Mura, Giuseppe Maccaferri, Mauro Nanni, Monia Negusini, Federico Perini, Mauro Roma, Matteo Stagni, Massimo Zucco, Davide Calonico

**Affiliations:** 1 Istituto Nazionale di Ricerca Metrologica, INRIM, Strada delle Cacce 91, 10135 Torino, Italy; 2 Istituto Nazionale di Astrofisica, Istituto di Radioastronomia, Bologna, Italy; 3 Rheinische Friedrich-Wilhelms-Universitat Bonn, Institut fur Geodesie und Geoinformation, Bonn, Germany; 4 Max-Planck-Institute for Radio Astronomy, Bonn, Germany

## Abstract

We describe a VLBI experiment in which, for the first time, the clock reference is delivered from a National Metrology Institute to a radio telescope using a coherent fibre link 550 km long. The experiment consisted of a 24-hours long geodetic campaign, performed by a network of European telescopes; in one of those (Medicina, Italy) the local clock was alternated with a signal generated from an optical comb slaved to a fibre-disseminated optical signal. The quality of the results obtained with this facility and with the local clock is similar: interferometric fringes were detected throughout the whole 24-hours period and it was possible to obtain a solution whose residuals are comparable to those obtained with the local clock. These results encourage further investigation of the ultimate VLBI performances achievable using fibre dissemination at the highest precision of state-of-the-art atomic clocks.

Since epochs, astronomy and time/frequency metrology are tightly related: in the past, timekeeping itself was actually based on astronomical quantities. However, in the sixties, the redefinition of the second in the International System of Units (SI), now based on atomic physics, determined a neat separation between the two fields. Even if astronomy, in particular in the radio domain, makes use of atomic clocks as timekeepers, the two sciences have progressed independently. The situation is changing nowadays: a deepest look into the Universe and a better knowledge of our planet require improved measurement capabilities and, as will be seen hereafter, this cannot neglect the latest progresses in metrology.

One of the most enabling techniques in radio astronomy is Very Long Baseline Interferometry (VLBI)^1^, which is based on the simultaneous observation of a radio sky with many antennas on the Earth surface. Correlation of the signals detected by distant antennas improves the angular resolution of the observations with respect to the single antenna. The magnification equals the ratio of the baseline, i.e. the distance between two antennas, and the single antenna dish diameter, when they are expressed in number of wavelengths of the VLBI receiving frequency, which determines the theoretically ultimate achievable resolution. It usually ranges from 10^4^ to 10^6^. VLBI is a powerful technique in many fields, from the study of compact radio sources to spectroscopy of the interstellar medium, which may disclose important findings in fundamental physics and in the search for dark matter. In addition, it is one of the most reliable techniques for Earth sciences, enabling, for instance, a better modelling of the Earth surface, of the atmosphere, of climate changes.

The signals of interest in VLBI range from 100 MHz to 300 GHz: the correlation of signals from distant antennas starts with down-conversion and sampling at each telescope. This is obtained using a local oscillator, whose frequency is referenced to an atomic clock. The spectral purity of the clock is of paramount importance, as in multiplication chains the phase noise scales as *N*^ 2^ (*N* being the ratio between the radio-signal and clock frequencies). For the same reason, the atomic clock must exhibit an excellent long-term stability: a loss in the coherence between clocks used in different antennas results in fading of the interferometer fringes.

So far, radio telescopes have been equipped with local atomic clocks, because it was impossible to receive a proper clock signal from a remote site such as a National Metrological Institute (NMI).

Today, time and frequency dissemination based on optical fibres^2^,^3^,^4^,^5^,^6^,^7^,^8^ allows transferring the stability and the accuracy of the most precise clocks without any degradation.

In this Letter, we describe a VLBI experiment where for the first time the clock reference is a remote standard delivered from a NMI using a coherent fibre link 550 km long. The set-up is permanent and paves the way for a complete investigation of the ultimate VLBI performances achievable using the dissemination at the highest precision of state-of-the-art atomic clocks.

## Time in VLBI observations

The radio astronomical observable is a time interval, and precisely the difference between the arrival times of the radio signal emitted by one source at different antennas. The observed delay *τ*_obs_ is the sum of different components, and in general we distinguish five main terms:


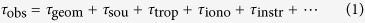


>where *τ*_geom_ is due to the geometry of the detection system, the position of the radio source, its distance and the position of the radio telescope; *τ*_sou_ accounts for the structure of the radio source (it is different if the signal is emitted by a compact or an extended celestial object), *τ*_trop_ and *τ*_iono_ are the atmospheric delays caused respectively by the troposphere and the ionosphere, namely the neutral and ionized part of the atmosphere; *τ*_instr_ is related to the instrumentation as a whole, including the antenna, cables, signal conditioning/acquisition and the timing reference.

The use of eq. 1 is manifold, in fact complex radio astronomical models for the delay and fitting of experimental data give access to the different components of the delay.

The most straightforward outcome of the model is the study of *τ*_sou_ that offers a direct access to the nature of distant radio sources. Another possible use is the investigation of *τ*_trop_, a relevant parameter for geophysics and atmospheric models.

A further relevant application is geodetic VLBI that uses known radio sources to determine the absolute value of the baselines, i.e. the distances among the antennas and to monitor their changes in time. The radio sources used in geodetic VLBI realize the International Celestial Reference Frame (ICRF) and this spatial reference is a fixed frame for geodesy. In geodetic VLBI, several distant antennas observe the signals from a set of sources each concurring to the realization of ICRF, so many parameters related to the source in eq. (1) are well known. The observations are composed of repeated campaigns each lasting 24 hours and followed by a correlation post processing that evaluates the length of the baselines and the single station coordinates.

Atomic clocks are crucial in such campaigns to offer the coherence needed for the data acquisition and to properly time tag the data for correlation. Atomic clocks performances are measured in terms of stability, namely the capability of reproducing a reference stable in time, and accuracy, the fidelity in the reproduction of the SI second. Therefore, a radio antenna has a time and frequency metrological chain that measures the frequency of the radio signal with respect to the local clock; traceability to the Universal Time Coordinated (UTC) is also important for a common time tagging of the data.

For the vast majority of geodetic and radio astronomical applications, the stability of the clock is among the most important features. In general, modern antennas use an active Hydrogen Maser (H-maser) as reference, which is the best compromise between cost and phase noise and time stability requirements. Active H-masers are commercial clocks used in many applications, starting from primary metrology, in particular in the realization of international reference timescales, hence also the UTC generation. Their instability is as low as 6 × 10^−14^ at 1 s in terms of Allan deviation of the relative frequency, while in the medium term they achieve a flicker floor typically between 3 × 10^−16^ and 1 × 10^−15^ in few hours. On the long term, a deterministic linear drift is present, ranging from few parts in 10^16^ to few parts in 10^15^, generally changing in time.

Currently, astronomy and radioastronomy deal with new observables like: fast radio bursts, extremely high-stability millisecond pulsars and in general a new class of experimental evidences of a very fast transient Universe. Even the recent detection of gravitational waves^9^ imposes to perform direct comparisons of atomic and astronomical observables, at an equivalent level of uncertainty, to get independent verifications and deeper insights into the fundamental physical properties of the Universe. All these challenges require a much higher level of timing resolution both at short and long timescales^10^. Then, it is now appropriate to explore new ways to cope with the need of better clocks.

The straightforward possibility is the upgrade of local clocks. Presently the short term stability is already improved using Cryogenic Sapphire Oscillators^11^ or photonic oscillators, for example at the ALMA radio telescope^12^, whilst in the near future both short term and long term stability would benefit from the new generation of highly accurate clocks, namely the optical clocks.

Nonetheless, there is another possibility that we address in this work and relies on the long-distance distribution of very stable and accurate signals. The common technique for remote clocks distribution uses satellites, in particular the Global Navigation Satellite Systems (GNSS) like the Global Positioning System (GPS) or the GLObal NAvigation Satellite System (GLONASS). The satellite methods transfer reference clock signals with a short term stability ranging from 10^−9^ to 10^−10^ at 1 s and achieving 10^−14^ to 10^−15^ over one day of operation. Therefore, radio astronomy cannot simply rely on satellite distribution; this is used just for traceability to UTC and for long-term control of the local maser performances. On the other hand, on hauls longer that 1000 km, optical fibre links have demonstrated an unprecedented stability of 10^−14^ to 10^−15^ in the very short term, i.e. few seconds^2^,^3^,^4^,^5^,^6^,^7^,^8^. Besides being widely exploited in frequency metrology to compare distant clocks, they offer new interesting possibilities for radio astronomy as well. Recently, this topic raised interest in the VLBI and frequency metrology community^13^,^14^. Firstly, fibre links permit the distribution of advanced clock signals directly from the NMIs, ensuring better performances than local H-masers. In particular, radio antennas can benefit from the constant improvement in the clocks performances pursued at NMIs. Also, different radio antennas can be time-referenced to a common clock using optical fibre, ensuring a partial rejection of the noise, a faster correlation and in general a better level of measurement, maybe also at a cheaper cost.

Finally, in the applications looking for accuracy, a direct access to UTC and to the SI second is possible.

## Experimental set-up

At the Italian NMI, INRIM, we realized the *Italian Link for Time and Frequency* (LIFT project), a fibre-based infrastructure for the distribution of frequency references to several research laboratories over the Country^3^. Among these, there is the Medicina radio observatory, managed by the National Institute of Astrophysics (INAF). It hosts two radio telescopes: the first Italian radio telescope, the *Northern Cross*, owned by the University of Bologna, and a 32-m parabolic dish. The station hosts also some geodetic facilities, namely two GNSS systems and a superconducting gravimeter, managed by the Italian Space Agency (ASI), the University of Bologna and the Bundesamt für Kartographie und Geodaesie (BKG, Germany), respectively.

The Medicina station cooperates to the European VLBI Network (EVN) and to the International VLBI Service for Geodesy and Radioastronomy (IVS) and is currently used both for single-dish and VLBI observations.

The optical link to Medicina is based on the distribution of a narrow-linewidth laser at 1.5 μm, which is phase-locked to a H-maser using an optical frequency comb. It is transmitted to Medicina through a phase-stabilized telecom fibre, which does not affect the uncertainty of the delivered frequency at the 10^−19^ level of precision^3^. In Medicina, an optical frequency comb is phase-locked to the fibre-delivered signal, and a microwave is synthesized as a harmonic of its repetition rate. If the comb parameters are appropriately chosen, this signal is at 100 MHz. A detailed description of the setup and its characterization can be found in ref. 13. Recently, this apparatus allowed us performing an absolute calibration of the atomic clock available at Medicina (an H-maser), with a precision exceeding the capabilities of GPS^13^. We now progress in this direction, demonstrating for the first time that the fibre-delivered signal is suitable as a local oscillator in VLBI observations and could replace the local H-maser.

The signal synthesized from the comb is processed and sent to a low-noise frequency divider, which generates two RF signals at 10 MHz and 5 MHz and a Pulse Per Second signal (PPS). Particular care has been devoted to the detection and processing electronics, to ensure that its instability does not affect the overall system performances. The PPS signal derived from this process has the same stability as the optical signal from which it is generated, but does not carry information about absolute time. Therefore, we synchronize it to a GPS-received PPS at the beginning of the measurement and after every possible loss of coherence. To efficiently compare the performances of the local clock and the fibre-delivered signal during VLBI campaigns, we set up an electronic system which is capable to switch between the two. VLBI does not strictly require any synchronization between the telescopes: in fact, it is possible to retrieve the single station timings at the ps level as a product of data analysis, as will be detailed in the following section. However, in practice, a rough *a priori* synchronization (at a level of hundreds of ns) is performed between various antennas relying e.g. on the GPS, as it greatly reduces the processing time during data analysis.

**Figure 1:**
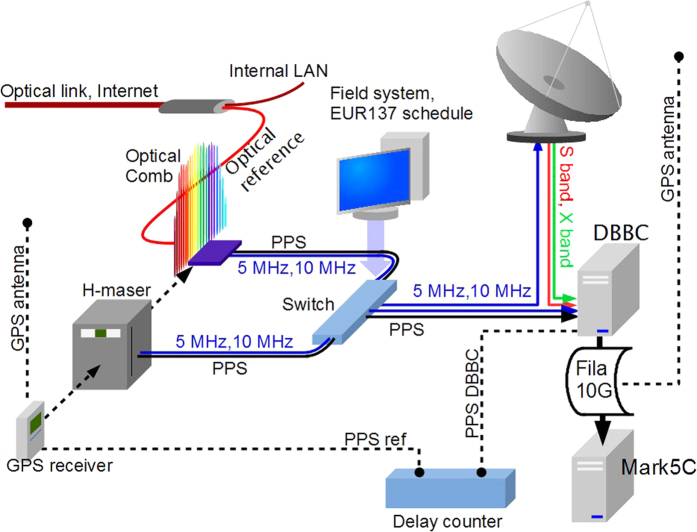
The experimental set-up with local and remote clock, generated by an optical comb referenced to the optical link signal; the sampling equipment Digital Base Band Converter (DBBC); the data formatting unit (FILA10G) and the Mark5C recording unit. The PPS signals generated with both clocks are slaved to GPS and the switch is controlled via software. Resynchronization of DBBC and FILA10G after each switch is provided by another GPS antenna and monitored on a time-interval counter.

The set-up used for the experiment is shown in Fig. 1. It is composed by the local and remote H-maser time/frequency references with the switching unit, the Field-System controlling software and the standard VLBI backend. The long term correction of the local H-maser is obtained through a dedicated GPS receiver (on the left in Fig. 1) which also provides the initial synchronization of the reference PPS signal generated by the local H-maser. This latter signal is also used for the initial synchronization of the PPS synthesized from the fibre link signal at the beginning of the experiment and after every possible loss of the coherence.

The VLBI backend is composed by a sampling equipment Digital Base Band Converter (DBBC), a data formatting unit (FILA10G)^15^ and a recording unit (Mark5C)^16^. The switch between the two references is controlled through the switch-box driven by the observation schedule, when an ad-hoc procedure, inserted in the original schedule, is called. Since the reference switching breaks the DBBC synchrony, it is necessary to perform a resynchronization of DBBC and FILA10G after each switch. To do that in an effective way, an external GPS signal has to be provided to FILA10G (on the right in Fig. 1) just for this purpose. In future experiments, this will be replaced by a time signal disseminated from INRIM via the fibre link.

## The EUR137 Experiment and Data Analysis

The IVS is an international collaboration of organizations, which operates and supports VLBI, provides a service to support research and operational activities, helps in the coordination of the experiments and provides data and products for the scientific community^17^. One of the tasks is to coordinate the observing program, which consists of various series of 24-hour observing sessions. One of these programs is called Europe, which involves the European radio telescopes and aims at determining the intra-European plate stability and tectonically induced strain accumulation. In order to test the remote clock, we decided to use the already planned Europe experiment (EUR137) scheduled on September, 7th 2015. We used both the fibre link and local H-maser standards throughout the same experiment. We did not split the session into two consecutive sub-sessions, but we alternated scans using both clocks.

**Figure 2:**
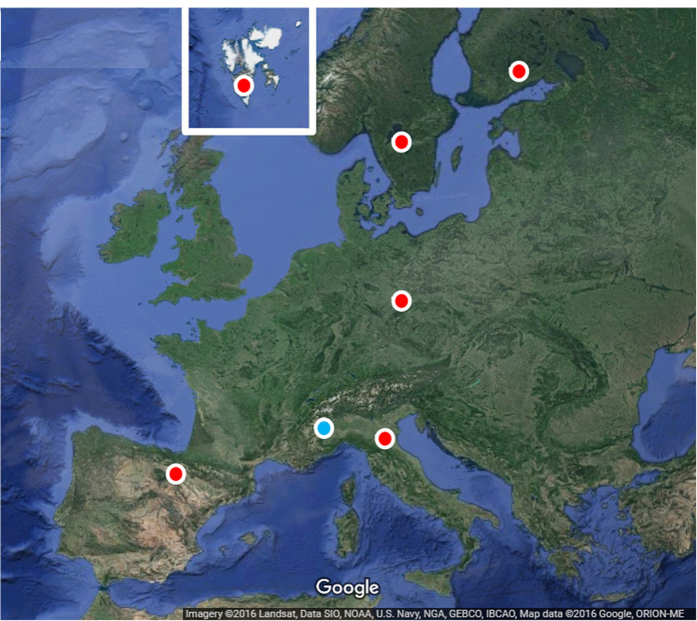
A map of the stations involved in the IVS EUR137 experiment. Red circles indicate the VLBI radio antennas; in blue, the position of the Italian National Metrology Institute. (Imagery ©2016 Landsat, Data SIO, NOAA, U.S. Navy, NGA, GEBCO, IBCAO, Map data ©2016 Google, ORION-ME)

The data were recorded at six European stations, namely DSS65 (Spain), MEDICINA (Italy), METSAHOV (Finland), NYALES20 (Norway), ONSALA60 (Sweden), WETTZELL (Germany) (Fig. 2). The experiment was correlated using the Bonn DiFX correlator^18^. Since for this experiment MEDICINA station was not scheduled, we decided to add it to the official schedule in the so called tag-along mode. This consists in simply adding the station to the schedule without changing the geometry of the observation, hence not changing the official geodetic output. At the moment of the correlation, we treated the observations performed at Medicina with the two different clocks as two different VLBI stations: MEDICINA and MEDILIFT. This gave us the opportunity to compare the behaviour of the system in real and comparable conditions.

**Figure 3:**
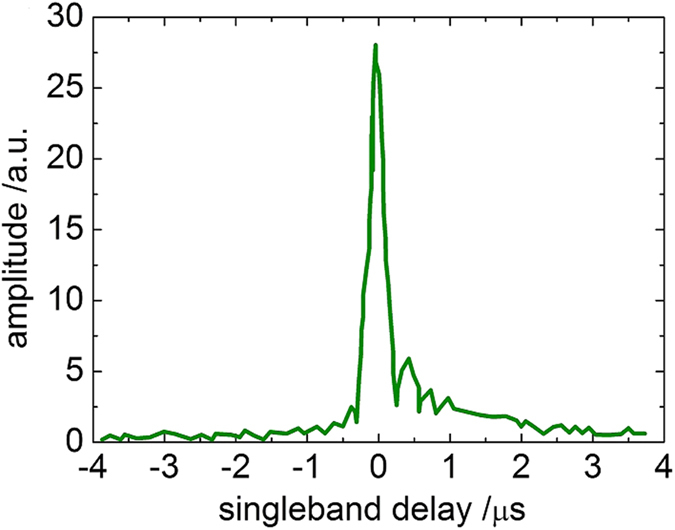
Correlation fringes on the baseline MEDICINA-WETTZELL for the EUR137 experiment.

The DiFX correlator output can be manipulated by the Haystack Observatory Postprocessing System^19^ that comprises *fourfit*, the program used to perform basic fringe-fitting, and operates between the correlator and an image-processing package. The fourfit plots show three windows related to multi band delay, single band delay, and averaged power spectrum. The main reference when searching for fringes is the single band delay window: when the fringe is not shown as 0 μs delay, it is necessary to adjust all the clocks delays and the clocks frequency drifts with respect to the reference antenna. This procedure can be viewed as a post-processed synchronization of all the antennas involved in the experiment. The multi band delay window shows the delay across the base band channels and it determines the overall quality of the observation scan. The power spectrum window shows the amplitude and phase of the full bandwidth observed divided in lower and upper side bands. As an example, we show in Fig. 3 the single band delay from the correlation between MEDILIFT and WETTZELL: the plot shows the correlation fringes detected on that baseline during a single scan. Once fringe fitting is performed and the data quality has been positively evaluated, the program converts correlated data into a dataset that can be handled with Mark-5 VLBI Analysis Software *Calc/Solve*^20^.

Two databases were created by *Dbedit* routine, for both S and X bands. Two frequencies observations are needed to deal with ionospheric delay. A priori physical models and information regarding stations, sources, Earth Orientation Parameters, loadings, meteorological and antenna information were added. These preliminary databases were analyzed with the geodetic VLBI software *νSolve*^21^. Both databases contain all the observations recorded throughout the 24-h session; generally, not all of them are used to obtain the solution, because of the Quality Code threshold, technical problems at the stations, not simultaneous observations recorded at both bands and so on. During the first steps of the analysis, single band delays are processed and clock offsets and rates are computed with respect to a reference station. Later, the group delays are analyzed, correcting for ionosphere delays, detecting outliers, to finally obtain a full solution. The residuals after fitting are computed for all the stations involved in the experiment using a full parametrization: for clocks a 2nd degree polynomial + 1-h Continuous Piece Wise Linear Fit functions are used, except for the reference station; for troposphere 1-h Zenith Wet Delay (ZWD) parameters and 24-h horizontal tropospheric gradients are computed; the station coordinates, except for a reference station, are estimated for the whole session, based on the complete set of observations, as well as the difference between Universal Time and Universal Time Coordinated and nutation offsets.

**Figure 4:**
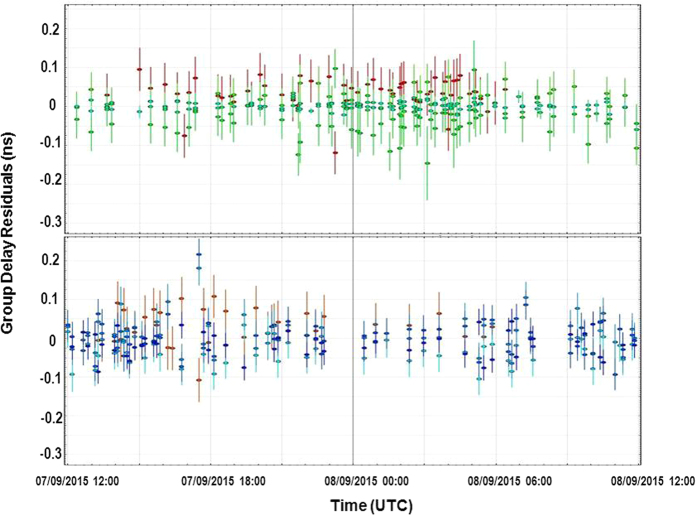
Residuals of the solution using MEDICINA (top) and MEDILIFT (bottom). Different colours indicate various baselines. The interaction effect of speed and dot periodicity. The F contrast of the interaction effect between speed and dot periodicity revealed no significant activation.

The residuals of the computed solutions using MEDILIFT and MEDICINA are compared in Fig. 4. The MEDILIFT residuals show a reliable Chi-square value and a Weighted Root Mean Square (WRMS) comparable to MEDICINA.

The quantitative analysis is reported in Tables 1 and 2.

Table 1 shows the WRMS of the delay residuals comparing the baselines between MEDICINA or MEDILIFT and the other stations. The MEDILIFT observations are comparable or slightly better than the other stations in 3 cases, for the baselines with DSS65A, METSAHOV and NYALES20. Considering ONNSALA60 and WETZELL, the WRMS of the residuals at 24 hours are respectively three and four times those using MEDICINA.

Table 2 shows the WRMS of the residuals for each station combining the data of all the possible baselines. The number of observations useful for the analysis in each station is 98% but for NYALES20 and MEDILIFT, for which the rate of useful data is 88% (259/296 observations). The 12% missing data is due to losses of the phase-coherence in the fibre transfer system, that are instrumentation-related and will be eliminated in a future upgrade of the instrumentation. Few issues with the front-end acquiring the RF synthesized from the link-delivered signal are presently under investigation. The overall result for MEDILIFT exhibits a larger WRMS value with respect to MEDICINA. If we consider only the first 5 hours of observations, as shown in Table 2, before a planned resynchronization of the remote clock, the MEDILIFT residuals exhibit a higher stability, comparable to the MEDICINA ones. After that break, many other jumps were present in the data and it was more complicated to resolve ambiguities in order to obtain a full solution for MEDILIFT. These effects are currently under investigation and will be addressed by future campaigns.

## Conclusion

In this Letter, we have demonstrated the realization and the in-field use of a permanent facility for primary time and frequency dissemination from the Italian Metrological Institute to the radio telescope of Medicina, Italy, at a distance of 550 km. We used the infrastructure in a real VLBI experiment, EUR137, where in 24 hours the local H-maser was alternated with the remote clock disseminated through the fibre link.

The reliability of the developed infrastructure is already satisfactory: the amount of data useful for analysis was nearly 90%, which can in principle be increased up to nearly 100% by a future technical upgrade of the set-up. The results obtained with the fibre-delivered clock show a good quality of the radio-astronomical observations throughout the entire 24-hours session; interferometric fringes were detected during the whole experiment, and it was possible to retrieve a solution for MEDILIFT whose residuals are comparable to those obtained with the local clock. These results clearly indicate that fibre frequency dissemination is a viable alternative to local clocks at radiotelescope sites and that further investigation is promising. The two reference chains will now be extensively tested during more circumscribed campaign using the Italian antennas in Medicina and in Noto (Sicily), as well as in other international schedules.

The further step is also to use an improved frequency standard, such as an optical clock^22^, in particular in the case of MEDILIFT, the Yb optical clock developed at INRIM. The motivation for this upgrade is twofold. Firstly, the coherence of a hydrogen maser is not appropriate for higher frequency observations in the range from 80 GHz to 300 GHz. With better clocks, radio astronomy can achieve the angular resolution needed to investigate compact radio sources or can directly study molecular emissions from the interstellar medium, which has an interest also in fundamental physics, such as general relativity and the quest for dark matter and dark energy. VLBI in this spectral region is known as mm-VLBI and is already present in Europe, USA, Mexico and Korea through the Global mm-VLBI Array^23^,^24^, whilst another important mm-radio astronomical antenna array is ALMA in Chile^25^.

In particular, much higher sky frequency acquisitions from very dry locations in appropriate observation strategies and/or direct observations of special astronomical objects with dedicated data acquisition systems and exploiting all possible timing capabilities can be expected to lead to new discoveries in fundamental physics and even in the metrology field. In addition, present H-masers are a limitation especially in forthcoming geodetic VLBI. Since few years, when the requirements for the next generation VLBI system were recommended, the IVS has addressed that the 1-mm accuracy level in geodetic campaign cannot be achieved using the present level of stability at the radio antenna facilities. Better clocks, such as optical frequency standards, would be beneficial for a further improvement of the geodetic model of our planet, geomonitoring and geophysical models^1^,^10^.

## Additional Information

**How to cite this article**: Clivati, C. *et al*. A VLBI experiment using a remote atomic clock via a coherent fibre link. *Sci. Rep.* **7**, 40992; doi: 10.1038/srep40992 (2017).

**Publisher's note:** Springer Nature remains neutral with regard to jurisdictional claims in published maps and institutional affiliations.

## Figures and Tables

**Table 1:** Delay residuals for all the possible baselines including MEDICINA or MEDILIFT.

Baseline	WRMS Residuals (ps) MEDICINA	WRMS Residuals (ps) MEDILIFT
DSS65A	50.7 (0.9)	45.3 (0.8)
METSAHOV	31.9 (0.7)	33.4 (1.0)
NYALES20	50.2 (0.98)	43.0 (1.0)
ONSALA60	13.6 (1.3)	35.8 (0.9)
WETTZELL	8.5 (1.1)	36.4 (0.9)

**Table 2:** Fraction of used observations (Used Obs.) and delay residuals (Res.) for each station, combining the data from all the possible baselines; columns 2 and 3 consider the whole dataset; columns 4 and 5 consider a dataset where for MEDILIFT only the first 5 hours of measurement are taken into account.

Station	24 h Used Obs.	24 h Res. (ps)	MEDILIFT 5 h Used Obs.	MEDILIFT 5 h Res. (ps)
DSS65A	744/756	26.2 (1.1)	719/756	21.7 (0.9)
MEDICINA	307/317	13.6 (1.0)	305/317	17.3 (0.9)
MEDILIFT	259/296	37.5 (0.9)	88/296	21.1 (0.9)
METSAHOV	866/884	29.3 (1.1)	834/884	28.1 (0.9)
NYALES20	827/943	28.4 (1.4)	889/943	26.0 (0.9)
ONSALA60	892/908	23.5 (1.1)	853/908	22.1 (0.9)
WETTZELL	971/994	21.7 (1.2)	930/994	21.8 (0.9)
